# Nephrotoxicity associated with aminoglycoside therapy in paediatrics: experiences from a leading referral hospital in Kenya

**DOI:** 10.1093/jacamr/dlae143

**Published:** 2024-09-05

**Authors:** Emmah Nyaboke, Anastasia Guantai, Margaret Oluka, Beatrice Mutai, Brian Godman, Amanj Kurdi, Marion Bennie, Mitchel Okumu

**Affiliations:** Department of Pharmacology and Pharmacognosy, School of Pharmacy, University of Nairobi, Nairobi, Kenya; Department of Pharmacology and Pharmacognosy, School of Pharmacy, University of Nairobi, Nairobi, Kenya; Department of Pharmacology and Pharmacognosy, School of Pharmacy, University of Nairobi, Nairobi, Kenya; Department of Paediatrics and Child Health, University of Nairobi, Nairobi, Kenya; Strathclyde Institute of Pharmacy and Biomedical Sciences, Strathclyde University, Glasgow, UK; Strathclyde Institute of Pharmacy and Biomedical Sciences, Strathclyde University, Glasgow, UK; Strathclyde Institute of Pharmacy and Biomedical Sciences, Strathclyde University, Glasgow, UK; Department of Medical Services, Public Health, and Sanitation, County Government of Kisumu, Kisumu, Kenya

## Abstract

**Introduction:**

This study assessed the prevalence and risk factors of nephrotoxicity in paediatric patients receiving aminoglycoside therapy at the Kenyatta National Hospital (KNH) in Kenya.

**Methods:**

Between July and September 2018, a prospective cohort study involving children receiving aminoglycoside treatment was carried out at KNH. Before beginning and after finishing the aminoglycoside therapy, the levels of serum creatinine were assessed. Descriptive statistics were used to describe the patients’ clinical and sociodemographic features. Associations between nephrotoxicity and maternal and paediatric variables were assessed using multivariable logistic regression.

**Results:**

The final analysis comprised 195 children and the prevalence of nephrotoxicity was 10.3%. Neonates made up 28.7% (58/195) of the total and their risk of developing nephrotoxicity was 3.54 (95% CI 1.6–8.21) times higher than that of other children (*P *=* *0.003). Neonates with low birth weight were 4.73 (95% CI: 1.8–12.5) times more likely to develop nephrotoxicity than those whose birth weight was >2500 g (*P *= 0.002). Neonatal patients with sepsis had a 4.91 (95% CI: 2.07–11.62) times greater association with acute kidney injury than neonates receiving treatment for other illnesses (*P *= 0.001). Sixty-five percent (13/20) of children who developed nephrotoxicity were switched to cephalosporins.

**Conclusions:**

Aminoglycosides were more nephrotoxic to asphyxiated, low-birth-weight neonates with sepsis. Routine monitoring of kidney function should be done within 72 h of starting aminoglycoside treatment in all neonates.

## Introduction

Aminoglycoside antibiotics are indicated for urinary tract, intra-abdominal and acute respiratory infections such as pneumonia, sepsis, meningitis in children, and skin and soft tissue infections.^1^ Drugs such as gentamicin and amikacin are aminoglycoside antibiotics, which are extensively used at the paediatric department of the Kenyatta National Hospital (KNH).^2^ They are inexpensive, accessible and generally well tolerated.^3–5^ However, their narrow therapeutic window and nephrotoxic potential are clinical concerns.^6–8^ Nephrotoxicity (defined as a rise in serum creatinine of ≥1.5 times the reference serum creatinine) is often mild and reversible.^2,5,9,10^ Factors that increase the risk of paediatric patients developing aminoglycoside-associated nephrotoxicity include age, neonatal sepsis, concurrent use of nephrotoxic medications, preterm delivery, birth asphyxia and low birth weight.^3,11,12^

A 2008 review by WHO reported that the prevalence of aminoglycoside-associated nephrotoxicity among children was between 1.2% and 55%.^3^ Moreover, acute renal injury was found in 20%–33% of children exposed to aminoglycosides in a recent epidemiological study using the RIFLE and KDIGO criteria.^5^ An audit on the use of aminoglycosides in children at KNH found a 5.8% incidence of aminoglycoside-associated nephrotoxicity and a 30% rise in serum creatinine.^2^ This incidence, however, might be greater given that the same study found that routine renal function monitoring was not carried out. To ascertain the true incidence of aminoglycoside-associated nephrotoxicity, this study expanded upon the previous one by doing routine monitoring of the renal function in this cohort. This study’s objectives were to identify the prevalence of aminoglycoside-associated nephrotoxicity in children under the age of 5 years who were admitted to KNH, as well as to describe the associated patient and maternal risk factors and the short-term outcomes of the patients receiving treatment.

## Materials and methods

### Ethics

The Ethics and Research Committee of the Kenyatta National Hospital/University of Nairobi critiqued this work and provided an approval (Ref. No. KNH-ERC/A/91).

### Study design and participants

#### Inclusion criteria

All children aged below 5 years on aminoglycosides and admitted to KNH between July and September 2018 were the subject of this hospital-based prospective cohort study. The neonatal units and general paediatric wards were used to sequentially recruit the individuals.

#### Exclusion criteria

Patients who began aminoglycoside therapy more than 3 h prior to baseline creatinine collection, those with underlying renal disease (serum creatinine levels of more than 70 µmol/L as a result of low muscle mass)^13^ and those admitted to oncology, renal and intensive care units and on aminoglycosides for greater than 3 h prior to baseline serum creatinine measurements were excluded from the study. Other exclusion criteria were patients who had been off aminoglycosides for more than 72 h and preterm newborns.

### Study outcome

The incidence of nephrotoxicity was the primary outcome of this study. It was determined using the RIFLE criteria and classified as a risk of injury when the serum creatinine levels were ≥1.5 times above the reference level, acute kidney injury (AKI) when the serum creatinine level increases by 2–3 times, or kidney failure when there was a ≥3 times increase in the baseline level of serum creatinine.

### Collection of blood samples

Before giving aminoglycosides or 3 h after giving the first dose of aminoglycosides (3 mg/kg/day for neonates less than 7 days old and weighing <2 kg, or 5 mg/kg/day for neonates more than 7 days old and weighing ≥2 kg), blood samples were taken to measure baseline serum creatinine levels. After completion of therapy, blood samples were obtained for the subsequent measurement of serum creatinine. Within 20 min of collection, the blood samples were delivered to the renal laboratory at room temperature (18–25°C). The levels of serum creatinine were calculated on the Cobas Integer device (Roche Cobas 8000 series). Creatinine and picrate interact in this device, causing the Jaffa reaction to give the mixture a crimson hue. When measured photometrically at 500 nm, the creatinine concentration directly relates to the intensity of the red colour that is produced.^14^

### Follow-up

Every day following the ward rounds, the clinical records were reviewed to check the progress of the children, and any pertinent updates regarding dose adjustments or discontinuation of aminoglycoside therapy were noted. Monitoring was continued when the aminoglycoside therapy was completed or when they switched to any other antibiotics. However, long-term follow-up of the patients who developed AKI was beyond the scope of this study.

### Data collection

While hospital records were used to collect data on the patients’ clinical characteristics, e.g. serum creatinine, mothers were interviewed to provide details on maternal characteristics (e.g. medical history, prior use of nephrotoxic medications during pregnancy). Factors such as birth weight, gestational age at birth, perinatal hypoxia, patent ductus arteriosus (PDA) or non-steroidal anti-inflammatory drug (NSAID) use were also documented. The admission histories, chronic illnesses, dehydration level and medication histories of children were also considered.

### Data analysis

STATA (v13.0, StataCorp., 2013)^15^ was used to analyse the collected data. Multivariate logistic regression analysis was used to evaluate the relationship between specific risk variables and aminoglycoside-associated nephrotoxicity.

## Results

### Demographic features of children on aminoglycoside therapy at KNH during the study period

A total of 195 children aged 5 years and under were recruited, with 93 (47.7%) being female and 102 (52.3%) being male. Their age ranged from 0.5 to 5 years, with 6 months being the median. Fifty-eight were neonates (29.7%), of whom 7 (12.1%) weighed less than 2500 g and 51 (87.9%) weighed more than 2500 g. The newborns had a mean gestational age of 38.2 ± 2.5 weeks.

### Clinical features

Thirty-one (31/195; 15.9%) of all children had previously been hospitalized, 28/195 (14.4%) were using medications for chronic illnesses at home, and 23 (11.8%) had previously used nephrotoxic drugs according to their prescription histories. Sixty-one (31.3%) of the children were dehydrated at admission. The median treatment time with amikacin was 3 days (IQR 3.0–4.0), whereas the median treatment time with gentamicin was 5 days (IQR 4.0–5.0). Thirty-six infants (18.5%) had neonatal sepsis, and 117 children (60%) had severe pneumonia as their primary diagnosis. Meningitis was found in 12 cases (6.2%), acute gastroenteritis in 8 cases (4.1%), perinatal asphyxia in 11 cases (5.6%) and various other disorders in 19 cases (9.7%). One hundred and sixty children received gentamicin (82%) and 35 children received amikacin (18%).

### Prevalence of aminoglycoside-induced nephrotoxicity, risk of kidney damage, and AKI

Twenty (20/195; 10.3%) of the children experienced nephrotoxicity and 12 (60%) of them were at risk of kidney damage. Five out of 195 (2.5%) developed AKI, while 3/195 (1.5%) exhibited kidney failure according to the paediatric RIFLE criteria, which define risk of injury as an increase in serum creatinine of ≥1.5 times the reference serum creatinine level or a decrease in the urine output to <0.5 mL/kg/h for >6 h, or AKI with a rise in serum creatinine by ≥2–3 times, and kidney failure with a rise by ≥more than 3 times the baseline serum creatinine.

### Clinical and demographic characteristics of mothers

The study comprised 195 mothers with an age range of 24–38 years and a mean age of 28.4 years. One hundred and eighty-one (181/195; 92.8%) of the participants were married, 125/195 (64.1%) were unemployed and 140/195 (70.8%) had completed high school. One hundred and nineteen of the mothers (92.8%) delivered their infants at KNH. Thirty (15.4%) of the mothers reported using NSAIDs at least once during their pregnancies, while 14 (7.2%) of the mothers had a history of admission during pregnancy. Most mothers (182/195; 93.3%) were healthy throughout their pregnancies; however; 6 (3.1%) of them had hypertension.

### Relationship between sociodemographic and clinical features of children and aminoglycoside therapy-induced nephrotoxicity

In comparison with children who weighed more than 2500 g, those who weighed less than 2.5 kg were 4.7 (95% CI: 1.8–12.5) times more likely to experience aminoglycoside-associated nephrotoxicity (*P *= 0.002). Neonatal infants younger than 28 days old had a 3.54 (95% CI: 1.6–8.21) times higher risk of developing aminoglycoside-associated nephrotoxicity than infants older than 28 days (*P *= 0.0032) (Table 1).

**Table 1.** The relationship between demographic and clinical features of children on aminoglycoside therapy with development of nephrotoxicity at KNH during the study period

Factor	Nephrotoxicity		RR (95% CI)	*P* value
	Yes (%)	No (%)		
Median age of the child (months)	0.4 (0.2–11.2)	6.3 (0.7–16.3)		0.238
≤28 days	12 (6.2)	46 (23.6)		
>28 days–2 years	6 (4.1)	101 (66.2)	3.54 (1.6–8.21)	0.003**
2 years–5 years	2 (0.1)	28 (14.4)		
Gender				
Male	10 (9.8)	92 (90.2)	0.91 (0.39–2.09)	0.827
Female	10 (10.8)	83 (89.2)		
Weight at birth				
<2500 g	3 (42.8)	4 (57.1)		
≥2500 g	17 (9)	171 (91)	4.73 (1.8–12.5)	0.002**
Dehydration				
Yes	6 (9.8)	55 (90.2)	0.94 (0.38–2.33)	0.896
No	14 (10.4)	120 (89.6)		
History of previous admissions				
Yes	1 (3.2)	30 (96.8)	0.27 (0.04–2.0)	0.204
No	19 (11.6)	145 (88.4)		
Concurrent nephrotoxic medication				
Yes	1 (4.3)	22 (95.7)	0.39 (0.1–2.8)	0.352
No	19 (11.0)	153 (89.0)		
Median duration of treatment (IQR)				
Amikacin	3.0 (3.0–3.5)	3.0 (3.0–4.0)	—	0.211
Gentamicin	5.0 (4.0–5.5)	5.0 (4.0–5.5)		
Aminoglycoside used				
Amikacin	2 (1.03)	32 (16.41)	0.52 (0.13–2.16)	0.367
Gentamicin	18 (9.23)	141 (72.31)		

Bold type indicates statistical significance (**P ≤ 0.05). RR, relative risk.

### Relationship between the underlying medical diseases and aminoglycoside-associated nephrotoxicity at KNH during the study period

Aminoglycoside-associated nephrotoxicity was 4.91 (95% CI: 2.07–11.62) times more likely to occur in neonates treated for neonatal sepsis than in neonates treated for other illnesses (*P *= 0.001). The relationship between the development of aminoglycoside-associated nephrotoxicity in the newborns and the other underlying diseases was not significant (Table 2).

**Table 2.** Relationship between underlying medical diseases and nephrotoxicity

Diagnosis	Nephrotoxicity		OR (95% CI)	*P* value
	Yes (%)	No (%)		
Neonatal sepsis (age ≤28 days)				
Yes	15 (25.8)	7 (12.1)	4.91 (2.07–11.62)	<0.001**
No	5 (8.6)	31 (53.4)	1.0	
Severe pneumonia				
Yes	9 (7.7)	108 (92.3)	0.5 (0.2–1.3)	0.154
No	11 (14.1)	67 (85.9)	1.0	
Meningitis				
Yes	0 (0)	12 (100.0)	0.345 (0.02–5.39)	0.448
No	20 (10.9)	163 (89.1)		
Acute gastroenteritis				
Yes	2 (18.2)	9 (81.8)	1.85 (0.49–7.01)	0.360
No	18 (9.8)	166 (90.2)		
Perinatal asphyxia in neonates (age ≤28 days)				
Yes	4 (6.9)	4 (6.9)	1.56 (0.70–3.48)	0.275
No	16 (27.6)	34 (58.6)		
Others				
Yes	0	11 (100.0)	0.3 (0.02–5.84)	0.484
No	20 (10.9)	164 (89.1)		

**Bold type indicates statistical significance (**P ≤ 0.05). RR, relative risk.

### Relationship between the sociodemographic and clinical features of mothers and aminoglycoside-associated nephrotoxicity in their babies

There was no significant relationship between maternal demographic and clinical features of the mothers and the children who experienced aminoglycoside-associated nephrotoxicity.

### Treatment outcomes in children on aminoglycosides over the short term

Before therapy, the mean body temperature for the 195 children was 39.8°C (SD: 0.25°C), while it was 36.5°C (SD: 0.68°C) after treatment. Thirteen (6.7%) of the children who developed aminoglycoside-associated nephrotoxicity were switched to cephalosporins. During the time of the study, no infant deaths were reported (Table 3).

**Table 3.** Short-term outcomes of the children treated with aminoglycosides at KNH

Treatment outcome	Developed nephrotoxicity, *n* (%)	
	Yes	No
Discharged	2 (1.0)	7 (39.5)
Switched to other antibiotics	13 (6.7)	122 (62.1)
Died	0 (0)	1 (0.5)
Continued treatment with dose adjustment	5 (2.6)	45 (23.1)

## Discussion

The incidence of aminoglycoside-associated nephrotoxicity in the population was determined to be 10.3% based on the RIFLE criteria. This result is in line with research conducted during the last 10 years, which revealed that rates of aminoglycoside-associated nephrotoxicity ranged from 3% to 35%.^10,12^ Moreover, a 5% prevalence of aminoglycoside-associated nephrotoxicity was identified in an earlier investigation at KNH.^2^ Being a newborn was significantly (*P* = 0.0032) associated with the development of aminoglycoside-related nephrotoxicity. This is because kidney function is not fully developed in newborns, making them more likely to suffer acute renal injury.^16^ In this population, using nephrotoxic medication raises the risk of kidney damage, especially if it is not taken carefully. An investigation of gentamicin use in a hospital in North America, for instance, revealed a 26.2% prevalence of aminoglycoside-associated nephrotoxicity.^5^ However, a Cochrane review of seven studies with 1321 newborn infants reported that nephrotoxicity was uncommon in North America.^6^ Moreover, no nephrotoxicity was reported in a study on 90 newborns on aminoglycosides for a mean of 9 days. This variance could be explained by the various definitions of renal damage.^17^

In our study, the aminoglycoside-associated nephrotoxicity was substantially correlated with the low birth weight of the neonates (*P *= 0.012). Similar studies have discovered that low-birth-weight infants had an AKI prevalence of between 34.5% and 79%.^1,18^ Low birth weight was not significantly associated with AKI, according to a study on the short-term effects of AKI in neonates with perinatal hypoxia at KNH.^19^

When compared with neonates who were treated for other disorders, those who had neonatal sepsis had a 13.29 (95% CI: 3.61–48.89) times higher risk of developing nephrotoxicity (*P *= 0.001). Additionally, sepsis has been demonstrated in published research to be a separate risk factor for the emergence of AKI. Fifty-two (26%) of the 200 term neonates with sepsis who Mathur *et al.* examined had AKI.^20^ In general, newborns have been demonstrated to be more susceptible to kidney injury than other children, particularly those with the risk factors mentioned. When administering aminoglycosides to this population, caution should be exercised. Daily monitoring, short-term use and extended dose interval regimens are advised.^21^

In our investigation, the nephrotoxicity shown in neonates younger than 28 days of age was not substantially correlated with perinatal hypoxia. Nevertheless, this stood in contrast to other published research that identified prenatal hypoxia as a risk factor for kidney damage in newborns, with a prevalence rate of between 7% and 72%. In a study conducted at KNH in 2006, asphyxiated newborns had an AKI frequency of 11.7%.^19^ However, the number of asphyxiated neonates was low (8; 13.8%) and as we did not base the sample size on an analysis of individual risk variables, more research with bigger sample sizes is needed to validate or refute our conclusion. Other underlying conditions such as acute gastroenteritis, meningitis, upper respiratory infections, and others did not significantly affect the children’s development of nephrotoxicity.

All 195 of the children received 24 h extended once-daily dosage. Similar studies have demonstrated that 24 hour dosing is just as effective as 12 hour dosing, with minimized toxicity, improved adherence and decreased bacterial failure.^7,8,22,23^ To lower the nephrotoxicity levels of this antibiotic, the extended daily dose could be incorporated into the prescribing guidelines for aminoglycosides.

The development of nephrotoxicity in the children did not substantially correlate with the maternal traits. According to some studies, a sizable sample size will be required to identify a relationship between the maternal traits and aminoglycoside-induced nephrotoxicity.^19,24^ However, other research revealed that mothers of infants with kidney damage took more medications while pregnant, particularly NSAIDs and antibiotics.^24,25^ In our study, we gathered thorough information on the drugs the mothers used while pregnant, as well as their admission history. We employed gentamicin (for aminoglycosides) and ibuprofen as the marker medication for NSAIDs. However, it is important to highlight that because we relied on the recall of the mothers to document the drugs that they had taken while pregnant, these data may be subject to recall bias. The medical history of pregnancy must be documented moving forward. Only the time when the patients were receiving treatment with aminoglycosides was monitored in this investigation. The body temperature returning to normal values shows that the treatment was successful. Of the patients who experienced nephrotoxicity, 5 (25%) continued to receive aminoglycoside treatment whereas 15 (75%) were moved to alternative antibiotics. Throughout the time of the study, there was no mortality in this group.

### Study limitations

The small sample size limited the identification of any correlations between maternal factors and development of nephrotoxicity in children. When the mothers were interviewed for information about their medical histories, some of them expressed confusion about the medications they had taken while pregnant. Finally, AKI is likely confounded by sepsis and severe infection. There was no way to identify whether new cases of AKI in these vulnerable patients was related to the systemic infection rather than the antibacterial exposure. Long-term follow-up of the patients who developed AKI was beyond the scope of this study. Moreover, delineating whether the incidence of aminoglycoside-associated nephrotoxicity was due to high levels and accumulation or levels were consequently high due to the administration of aminoglycosides was beyond the scope of this study.

### Conclusions

Neonates had a higher risk of developing nephrotoxicity than other paediatric patients. Those with sepsis and low-birth-weight newborns were also more likely to experience aminoglycoside-associated nephrotoxicity. Nephrotoxicity was not linked to maternal variables or other patient-related risk factors including prenatal hypoxia, meningitis or the length of treatment with aminoglycosides. In high-risk populations, such as neonates, especially those with low birth weight, hypoxia or neonatal sepsis, aminoglycosides are advised to be administered with caution. After 72 h of treatment, all neonates receiving aminoglycosides should have routine renal function testing done since they have an increased risk of developing aminoglycoside-associated nephrotoxicity. To provide future guidance, larger studies are required to correlate maternal variables and other patient-related risk factors, like prenatal asphyxia, with nephrotoxicity and to evaluate the long-term outcomes of the patients who develop aminoglycoside-associated nephrotoxicity.
